# Study of Bacterial Community Composition and Correlation of Environmental Variables in Rambla Salada, a Hypersaline Environment in South-Eastern Spain

**DOI:** 10.3389/fmicb.2018.01377

**Published:** 2018-06-21

**Authors:** Nahid Oueriaghli, David J. Castro, Inmaculada Llamas, Victoria Béjar, Fernando Martínez-Checa

**Affiliations:** ^1^Microbial Exopolysacharide Research Group, Department of Microbiology, Faculty of Pharmacy, University of Granada, Granada, Spain; ^2^Institute of Biotechnology, University of Granada, Granada, Spain

**Keywords:** biodiversity, bacteria, hypersaline habitat, Rambla Salada, DGGE, dilution-to-extinction methods

## Abstract

We studied the bacterial community in Rambla Salada in three different sampling sites and in three different seasons and the effect of salinity, oxygen, and pH. All sites samples had high diversity and richness (Rr > 30). The diversity indexes and the analysis of dendrograms obtained by DGGE fingerprint after applying Pearson's and Dice's coefficient showed a strong influence of sampling season. The Pareto-Lorenz (PL) curves and *Fo* analysis indicated that the microbial communities were balanced and despite the changing environmental conditions, they can preserve their functionality. The main phyla detected by DGGE were *Bacteroidetes* (39.73%), *Proteobacteria* (28.43%), *Firmicutes* (8.23%), and *Cyanobacteria* (5.14%). The majority of the sequences corresponding to uncultured bacteria belonged to *Bacteroidetes* phylum. Within *Proteobacteria*, the main genera detected were *Halothiobacillus* and *Roseovarius*. The environmental factors which influenced the community in a higher degree were the salinity and oxygen. The bacteria belonging to *Bacteroidetes* and *Proteobacteria* were positively influenced by salinity. Nevertheless, bacteria related to *Alpha-* and *Betaproteobacteria* classes and phylum *Firmicutes* showed a positive correlation with oxygen and pH but negative with salinity. The phylum *Cyanobacteria* were less influenced by the environmental variables. The bacterial community composition of Rambla Salada was also studied by dilution-to-extinction technique. Using this method, 354 microorganisms were isolated. The 16S sequences of 61 isolates showed that the diversity was very different to those obtained by DGGE and with those obtained previously by using classic culture techniques. The taxa identified by dilution-to-extinction were *Proteobacteria* (81.92%), *Firmicutes* (11.30%), *Actinobacteria* (4.52%), and *Bacteroidetes* (2.26%) phyla with *Gammaproteobacteria* as predominant class (65.7%). The main genera were: *Marinobacter* (38.85%), *Halomonas* (20.2%), and *Bacillus* (11.2%). Nine of the 61 identified bacteria showed less than 97% sequence identity with validly described species and may well represent new taxa. The number of bacteria in different samples, locations, and seasons were calculated by CARD-FISH, ranging from 54.3 to 78.9% of the total prokaryotic population. In conclusion, the dilution-to-extinction technique could be a complementary method to classical culture based method, but neither gets to cultivate the major taxa detected by DGGE. The bacterial community was influenced significantly by the physico-chemical parameters (specially the salinity and oxygen), the location and the season of sampling.

## Introduction

In hypersaline environments not only, the high salt concentration limits the biodiversity that inhabits them, they also have, depending on the geographical area, low oxygen concentrations, high or low temperatures, and sometimes alkaline conditions. In addition, factors like pH, pressure, low nutrient availability, solar radiation, the presence of heavy metals, and other toxic compounds, may influence their biodiversity (Ventosa, 2004; Bell and Callaghan, 2012). These environments can be thalassohaline or athalassohaline, the first ones have a marine origin and a qualitative composition similar to sea water. In the second ones, the salt composition is similar to the composition of the surrounding geology, topography, and climatic conditions; this is particularly influenced by the dissolution of mineral deposits (Oren, 2002). Extremely and moderately halophilic microorganisms (bacteria and archaea) predominate in hypersaline environments (Ventosa, 2006; Ventosa et al., 2008; Oren, 2011).

Cultivation-based methods are widely used but often in microbial communities *in situ*, the most abundant members cannot be detected (Rappé and Giovannoni, 2003). During the 1990s and throughout 2000s, the fields of molecular ecology and metagenomics have significantly advanced our knowledge of the genetic diversity and distribution of environmental bacteria. Many new candidate divisions of bacteria and archaea are now recognized due to 16S rRNA sequence-based approaches and environmental metagenomics (Curtis et al., 2002). The numerically dominant bacteria of soils and rhizospheres are the *Alpha*-, *Beta*-, and *Gammaproteobacteria, Actinobacteria, Acidobacteria, Verrucomicrobia, Planctomycetes, Bacteriodetes*, and *Firmicutes* (da Rocha et al., 2009). These findings reveal the vast disparity between the phyla now recognized by molecular methods and those with cultured representatives.

Novel cultivation strategies are addressing the problem of the uncultivable majority of bacteria and have led to resurgence in microbial cultivation. Emerging strategies roughly follow four major lines: (1) Reformulated and improved culturing media employ dilute nutrient media, non-agar matrices, alternative electron receptors and donors, increased incubation times, and modified atmospheres similar to the bacterial environment (Joseph et al., 2003; Schoenborn et al., 2004; Davis et al., 2005). (2) Diffusion chambers grow bacteria in simulated natural environments (Ferrari et al., 2005, 2008; Gavrish et al., 2008; Ferrari and Gillings, 2009). (3) Microbial signaling molecules are added to growth media that replace natural signaling molecules essential for formation of biofilms and natural microbial consortia (Bruns et al., 2003; Stevenson et al., 2004). (4) High throughput cell separation methods employ either fluorescence activated cell sorting (Zengler et al., 2002, 2005) or dilution-to-extinction (Connon and Giovannoni, 2002) to separate individual bacterial cells to initiate enrichment cultures in dilute natural media.

In order to study the microbial populations in complex habitats has been used several combinations of molecular techniques (Oren, 2003, 2007) among them, the denaturing gradient gel electrophoresis (DGGE) (Muyzer and De Waal, 1994; Muyzer et al., 1996) together with the catalyzed reporter deposition-fluorescence *in situ* hybridization (CARD-FISH) (Wagner et al., 2003; Amann and Bernhard, 2008). Regarding to the use of independent culture techniques, several authors have studied the prokaryotic diversity in athalassohaline habitats, such as the lake Tebenquiche at Salar de Atacama in Chile (Demergasso et al., 2008), lakes of mountains in the Tibetan plateau (Wu et al., 2006), hypersaline alkaline lakes, such as the Mono lake in California (Humayun et al., 2003) and Wadi An Natrun in Egypt (Mesbah et al., 2007), alkaline evaporation ponds at Sua Pan in Botswana (Gareeb and Setati, 2009), Chott El Jerid, a Tunisian hypersaline lake (Abdallah et al., 2016), saline-alkaline soil located in Ararat Plain (Armenia) (Panosyan et al., 2018) and also in different solar salterns, such as Çamalti Saltern, the biggest artificial marine solar saltern in Turkey (Mutlu and Güven, 2015).

Rambla Salada is a clear example of athalassohaline habitat located in Murcia (south-eastern Spain) with special interest by the European Union and it has been declared as a protected wildfowl zone by its regional government (BORM 10/09/1998). Rambla Salada is a course of ~27 km that connects two areas of great ecological significance, the Natural Park of Sierra Espuña and the river Segura. It is based on an extensive area of sedimentary materials in which underground water emerged, together with the low rainfall, originates a number of wadis and streams. The salinity in Rambla Salada is due to Miocene evaporitic, gypsiferous and marly deposits (Muller and Hsü, 1987) and the most abundant of ions are Na^+^ and Cl^−^, followed by ${\text{SO}}_{4}^{2-}$ and Ca^+2^ (Ramírez-Díaz et al., 1995). This habitat has been widely studied by our research group since 2005, and five novel halophilic bacterial species have been described so far: *Idiomarina ramblicola* (Martínez-Cánovas et al., 2004), *Halomonas cerina* (González-Domenech et al., 2008), *Halomonas ramblicola* (Luque et al., 2012a) *Blastomonas quesadae* (Castro et al., 2017), and recently *Roseovarius ramblicola* (Castro et al., 2018). We have also described its halophilic archaeal community by molecular (Oueriaghli et al., 2013) and classical culture techniques (Luque et al., 2012b), its cultivable halophilic bacteria (Luque et al., 2014) and the distribution of *Halomonas* species by molecular methods (Oueriaghli et al., 2014).

In this work we used the DGGE technique, a culture-independent method, to study the diversity of the bacterial population and to establish the relationships with environmental variables such as pH, oxygen, salinity, and temperature using multivariate statistical analysis and we compare the diversity obtained by this method with those obtained using cultivation methods and incubation conditions including standard culture media (Luque et al., 2014) and dilution-to-extinction method, a technique that improves slow-growing microorganisms recovery or microorganisms that are apparently uncultivable. This unusual comparison in ecological studies enhances the importance of our study. The bacterial community by molecular methods was also quantified.

## Materials and methods

### Sampling sites description

The samples were taken from each of three different points in Rambla Salada at three different periods: June 2006, February, and November 2007. We took 6 samples during each sampling period, 18 samples in total: at Finca La Salina, the samples consisted of soil and watery sediment, from area next to the river (riverbed zone); watery sediment from the water-transfer conduit between the Tagus and Segura rivers (transfer zone) and watery sediment from a saline groundwater spring (upwelling zone) as shown in Table 1. The samples obtained from riverbed and transfer zone showed less salinity than those obtained in the upwelling zone. The salt concentration was higher in June 2006, a season characterized by low rainfall.

**Table 1:** Physico-chemical parameters at the three sites and sampling seasons in which samples were taken.

**Sites**	**Co-ordinates**	Sample^*^	**Season**	**Physico-chemical parameters**		
				**O_2_ (mg L^−1^)**	**Salinity (g L^−1^)**	**pH**
Site 1: Riverbed zone	38° 07′ 34.44″ N; 1° 07′ 11.13″ W	S1A, S2A	June, 2006	10.2	44.4	6.3
		S4A		4.5	83	6.8
		S1B, S2B	February, 2007	17.2	18.6	8.2
		S4B		14.3	20.2	8.1
		S1C, S2C	November, 2007	13	11.8	8.3
		S4C		7.9	22.2	8.0
Site 2: Transfer zone	38° 07′ 30.23″ N; 1° 07′ 42.22″ W	S3A	June, 2006	1.5	62.1	8.7
		S3B	February, 2007	10.5	34	8.1
		S3C	November, 2007	20.6	29	8.3
Site 3: Upwelling zone	38° 07′ 29.09″ N; 1° 07′ 42.15″ W	S7A, S8A	June, 2006	1	140	7.1
		S7B, S8B	February, 2007	0.6	157.6	6.7
		S7C, S8C	November, 2007	4	151.2	7.2

*Data from Luque et al. (2012b); Oueriaghli et al. (2013, 2014)*.

* *Type of sample: S1 and S2, soil sample; S3, S4, S7, and S8 watery sediments*.

The watery sediments were taken from the top 15 cm of the silt deposits in each sampling point. The samples were stored in sterile polycarbonate tubes and immediately taken to the laboratory, where they were stored at 4°C until study, always within 24 h. We determined *in situ* pH, oxygen, temperature, and conductivity at each sampling site using an ECmeter (TetraConR 325), which automatically calculates salinity.

### Low-nutrient medium

We used S3, a low-nutrient medium (Sait et al., 2002, 2006) supplemented with 3 and 15% (w/v) sea-salt solution (Rodriguez-Valera et al., 1981) and pH adjusted to 5.5, 7, and 10. The composition of the medium is the follow: sea-salt stock 30% (w/v) (Rodriguez-Valera et al., 1981), proteose peptone (0.5 g), trace element solution^†^ (2 ml), vitamins solution I^‡^ (2 ml), vitamins solution II^$^ (6 ml), selenite/tungsten^*^ solution (2 ml), purified agar (20 g), DI H_2_0 (1,000 ml).

^†^Trace element solution: HCl 25% (10 ml), FeCl_2_ x 4H_2_O (1.5 g), CoCl_2_ x 6H_2_O (190 mg), MnCl_2_ x 4H_2_O (100 mg), ZnCl_2_ (70 mg), H_3_BO_3_ (6 mg), Na_2_MoO_4_ x 2H_2_O (36 mg), NiCl_2_ x 6H_2_O (24 mg), CuCl_2_ x 2H_2_O (2 mg), DI H_2_O (1,000 ml).

^‡^Vitamins solution I: 4-aminobenzoate (40 mg), Biotin (10 mg), hemicalcium D+/–pantothenate (100 mg), pyridoxamine hydrochloride (50 mg), thiamin hydrochloride (150 mg), cyanocobalamin (100 mg), DI H_2_O (1,000 ml).

^$^Vitamins solution II: nicotinic acid (33 mg), DL-6,8-thioctic acid (10 mg), riboflavin (10 mg), folic acid (4 mg), DI H_2_O (1,000 ml).

^*^Selenite/tungstate solution: NaOH (0.5 g), Na_2_SeO_3_ x 5H_2_O (3 mg), Na_2_WO_4_ x 2H_2_O (4 mg), DI H_2_O (1,000 ml).

### Dilution-to-extinction method

The cultivation method used in this work was based on the dilution-to-extinction approach (Button et al., 1993; Connon and Giovannoni, 2002; Bruns et al., 2003; Koch et al., 2008). For this purpose, serial dilutions from 1 g of soil and/or 1 ml of watery sediment were prepared; soil samples were previously sonicated for 30 s, in 10 ml of S3 medium. The number of microorganisms in each dilution was determined using a Petroff Hausser counting chamber using methylene blue as contrast. A 48-well microtiter plate, containing 490 μl of supplemented S3 medium, was inoculated with 10 μl of the dilution which containing 100 bacteria per milliliter (~1 bacterium per well) and incubated at 25°C for 30 days. The bacteria grown in the wells were then isolated in Difco™ R2A agar medium plates (Reasoner and Geldreich, 1985) supplemented with 3 and 15% (w/v) sea-salt solution (Rodriguez-Valera et al., 1981).

### DNA extraction and partial bacterial 16S rRNA gen amplification

Total DNA was extracted, within 24 h since the samples were taken, from 10 g of each of samples using the PowerMax™ Kit for Soil (MO BIO Laboratories) according to the manufacturer's instructions. The primers used for the variable region amplification V1–V3 (~500 bp) of the 16S rRNA gene of domain *Bacteria* were Bact-8F (5′ AGAGTTTGATCCTGGCTCAG 3′) (Edwards et al., 1989) and the reverse primer Bact-518R (5′ ATTACCGCGGCTGCTGG 3′) (Muyzer et al., 1993). A 40-bp-long GC clamp (5′CGC CCG CCG CGC CCC GCG CCC GTC CCG CCG CCC CCG CCC G-3′) was attached to the 5′ end of the forward primer to obtain PCR fragments adequate for DGGE analysis (Muyzer et al., 1996). PCR reactions were carried out as described by Oueriaghli et al. (2013). An electroforesis in a 1.5 % w/v agarose gel in TBE 1 × buffer was used to separate the PCR products (5 μl). Then, the DNA bands were concentrated using Amicon Ultra 0.5 ml 100 K centrifugal filters (Eppendorf, Hamburg, Germany).

DNA from pure culture strains isolated by dilution-to-extinction methods were extracted with X-DNA Extraction Kit from XtremBiotech S.L. (www.xtrembiotech.com) according to the protocol provided by the company. In this case, PCR amplification of 16S rRNA gene was performed as described elsewhere (Castro et al., 2017).

### DGGE

A universal mutation detection system, Bio-Rad DCODE™, was used to carry out the denaturing gel gradient electrophoresis. “A 45 to 60% (w/v) (7 M urea and 40% deionized formamide) in a gel with 8% w/v polyacrylamide (37.5:1 acrylamide/bisacrylamide) was used. Each sample (1,000–1,600 ng of PCR products) was loaded onto the gel and run for 20 min at 200 V and again at 100 V at 60°C for 16 h in 1 × TAE buffer. The DGGE gel was stained with a 1:10,000 dilution of a stock solution of Syber® Gold (Invitrogen-Molecular Probes) for 45 min. DNA bands were visualized with an UV transilluminator (Molecular Imager®, Gel Doc™ XR System, Bio-Rad). All the type bands were excised from the gel, but among those at the same level, only three were chosen at random for cutting. They were resuspended in 10 μl of Milli-Q water and kept overnight at 4 °C. An aliquot of 2 μl of the supernatant was reamplified using the original set of primers (Bact-8F without the GC clamp and Bact-518R) under the conditions described above. The PCR products were purified with Illustra® GFX DNA before being sequenced with an ABI PRISM dye-terminator, cycle-sequencing, ready-reaction kit (Perking-Elmer), and an ABI PRISM 377 sequencer (Perking-Elmer) according to the manufacturer's instructions” (Oueriaghli et al., 2013).

### Phylogenetic study

The variable V1–V3 region sequences of the 16S rRNA gene obtained from DGGE and almost complete sequences of the 16S rRNA gene from dilution-to-extinction isolates were compared using BLASTN program (Altschul et al., 1997) against the GenBank/EMBL/DDBJ database to determine their phylogenetic affiliations. The sequences were then aligned using ClustalW included in MEGA 7 software (Kumar et al., 2016). The phylogenetic relationships of sequences obtained from the DGGE bands with those from the databases (more than 90 % identity) was studied applying neighbor-joining (NJ), maximum likelihood (ML), and maximum parsimony (MP) methods using MEGA 7. The phylogenetic trees and their robustness were tested by bootstrap analysis with 1000 replicates. *Aquifex pyrophilus* Kol5a^T^ (M83548) was used as out group.

### DGGE fingerprint analysis

FPquest v.5.101 software (Bio-Rad®) was used to standardize and compare the DGGE band patterns and clustering analysis was performed by determining the Pearson and Dice coefficients. Pearson coefficient takes into consideration the intensity of each band, and the Dice coefficient is based on the presence or absence of bands. Dendrograms relating band-pattern similarities were automatically calculated with UPGMA algorithms (unweighted pair-group method with arithmetic mean). The significance of UPGMA clustering was estimated by calculating the cophenetic correlation coefficients (Sokal and Rohlf, 1962).

### Diversity indexes

Data derived from the presence/absence of bands and from their intensity were exported from the FPquest program to determine the corresponding indexes. We calculated the Shannon-Weaver H' (diversity) and Simpson SI' (dominance) indexes (Shannon and Weaver, 1963; Magurran, 1996) for each DGGE lane using the following equations:

$$\begin{array}{l} H\prime =\sum_{i\;=\;1}^{s}pi\;ln\;pi\;\;\;SI\prime ={\sum \left(pi\right)}^{2} \end{array}$$

*S* corresponds to the total number of bands in a DGGE lane and *pi* is calculated as *pi* = *ni* / *N*; *ni* is the intensity of each individual band and *N* the sum of the intensities of all the bands in the analized DGGE lane.

Using the equation *Rr* = (*N2* × *Dg*) we estimated range-weighted richness index (*Rr*) where *N* represents the total number of bands in each DGGE pattern and *Dg* is the denaturing gradient between the first and last band of each pattern (Marzorati et al., 2008). As described in our previous work (Oueriaghli et al., 2013), “the evenness of the bacterial community was represented graphically by using the Pareto-Lorenz (*PL*) distribution curves on the basis of the DGGE fingerprints (Marzorati et al., 2008) and the bands in each DGGE lane were assorted according to their intensity. The cumulative normalized numbers of bands are represented along the *x*-axis and their respective cumulative normalized intensities are represented along the *y*-axis. The 45° diagonal represents the perfect evenness of a community in which all the species are equally abundant. To interpret the *PL* curves numerically and calculate the functional organization index of evenness (*Fo*) we plotted the *y*-axis with the vertical 20% *x*-axis line (Marzorati et al., 2008). The resulting values indicate the percentage of total band intensities that constitute 20% of the population. STATGRAPHICS® *plus* v. 3.2 (STSC, Rockville, MD, USA) was used for the analyses of variance (ANOVA). A significance level of 95% (*p* < 0.05) was chosen.”

### Multivariate statistical analysis

The influence of environmental variables upon bacterial diversity was evaluated by applying the detrended correspondence analysis (DCA) (Lepš and Šmilauer, 2003). Also, according to Oueriaghli et al. (2013) “we applied a CCA analysis using CANOCO 4.5 (Biometris, Wageningen, Netherlands). A Monte Carlo test was used to determinate the significance of each axis and to evaluate the influence of the environmental variables upon the overall distribution of bacterial species and their distribution at each sites and sampling seasons. The significance of the CCA axes was tested by means of 999 unrestricted permutations in order to check the null hypothesis that the bacterial profiles were not related to the environmental variables. The effect of any determined environmental variable was chosen according to its significance level (*p* < 0.05) (Salles et al., 2004; Sapp et al., 2007). Ordination biplots are used to represent the effect of environmental variables on bacterial community structure. The environmental factors are represented as arrows: the length of the arrows indicates the relative importance of that environmental factor in explaining the variation in the bacterial communities, whilst the angle between each arrow and the nearest axis indicates the closeness of the relationship between each other.”

### CARD-FISH

Catalyzed reporter deposition (also known as tyramide signal amplification) *in situ* hybridization (CARD-FISH) was conducted to stain bacterial cells selectively (Pernthaler et al., 2002) and was carry out according to the protocol described by our group in a previous work (Oueriaghli et al., 2013). “The preparation of samples was done by suspending 0.5 g of soil or watery sediments or 0.5 ml of water in 10 ml of PBS buffer 1X. In the case of soil or watery sediment, the resulting suspension was sonicated using the Ultrasonic (Sonorex Digitec) system for 20 min. Supernatant (1 ml of each) were fixed overnight with paraformaldehyde (2%) at 4°C. Cells were filtered and immobilized on 0.2-μm pore-size filters (GTTP, Millipore, Eschobron, Germany), embedded in 0.1% w/v agarose and permeabilized by treatment with 10 mg/ml lysozyme in 50 mM EDTA and 100 mM Tris/HCl for 1 h at 37°C (Pernthaler et al., 2002). Filter sections were cut and hybridized with a mixture of 50 ng/μl of horseradish-peroxide-labeled oligonucleotide probe Eub338 (Amann et al., 1995) (2:20 for each section) and buffer hybridization (Pernthaler et al., 2002) for 2.5 h at 35°C. For signal amplification, we used fluorochrome-labeled tyramide (1 mg/ml; FITC) (Pernthaler et al., 2002). All the microbial cells were counterstained with 4′,6′diamidino-2-phenylindole (DAPI) at a final concentration of 1 mg/ml (Snaidr et al., 1997). For microscopy, filters were first embedded in Citifluor™ (Citifluor Ltd., London, UK), after which the cells were studied under a Leica TCS-SP5 confocal laser scanning microscope (CLSM). Controls with the antisense probe HRP-Non915 were always negative. CARD-FISH stained cells were counted in 20 randomly selected frames using ImageJ software (http://rsb.info.nih.gov/ij/) (Rhasband, 2010).”

## Results

### Dilution-to-extinction approach

Dilution-to-extinction culturing yielded 182 positives microtiter plate wells from a total of 4,800 inoculated wells from which we obtained 354 isolates after re-isolation in R2A medium plates. BLAST searches of the sequences in GenBank revealed that the strains belonged to *Proteobacteria* (81.92%), *Firmicutes* (11.30%), *Actinobacteria* (4.52%), and *Bacteroidetes* (2.26%) phyla with *Gammaproteobacteria* as predominant class (65.7%). Detailed phylogenetic analysis revealed that the isolates shared 94 to 100% 16S rRNA gene sequence identity with the most closely related validly described species. A total of 61 different species were isolated (Table 2). The most abundant taxa were *Marinobacter* (38.85%), *Halomonas* (20.2%), and *Bacillus* (11.2%). Nine of the 61 identified bacteria showed less than 97% sequence identity with validly described species and may well represent new taxa.

**Table 2:** Taxa isolated by dilution-to-extinction, identified by comparison of their 16S rRNA gene sequences using BLAST.

**Isolate**	**GenBank acc. No**.	**Closet relatives and acc. No**.
PL20	MH266125	*Acinetobacter albensis* ANC 4874 (NR_145641)
25-D-8	MH266126	*Alcanivorax jadensis* T9 (NR_025271)
M35R	MH266127	*Alkalibacillus silvisoli* BM2 (NR_041482)
SR54	MH266128	*Bacillus enclensis* SGD-1123 (KF265350)
D16_1	MH266129	*Bacillus halotolerans* ATCC 25096 (LPVF01000003)
D28_1	MH266130	*Bacillus safensis* kv2 (MH200636)
16SRL	MH266131	*Bacillus siamensis* KCTC 13613 (AJVF01000043)
R63L	MH266132	*Bacillus tequilensis* FJAT-40022 (MG905894)
SR43	MH266133	*Bacillus thioparans* KmS3200909 (MG011570)
M5-FL	MH266134	*Bacillus velezensis* CR-502 (AY603658)
D22-913	MH266135	*Blastomonas quesadae* 912 (KX990274)
R25L	MH266136	*Citreimonas salinaria* CL-SP20 (NR_043303)
SR37F	MH266137	*Erythrobacter litoralis* HMF8222 (KY047411)
D11	MH266138	*Erythrobacter longus* DSM 6997 (NR_041889)
2-C-1	MH266139	*Erythrobacter marinus* HWDM-33 (NR_109054)
D40-857	MH266140	*Flavobacterium jumunjinense* HME7102 (NR_109367)
M35(1)RL	MH266141	*Halobacillus alkaliphilus* SP22 (KX885464)
ML35R	MH266142	*Halobacillus halophilus* 3 (NR_075035)
M45	MH266143	*Halomonas alimentaria* YKJ-16 (NR_025054)
F-5-2	MH266144	*Halomonas fontilapidosi* HMF4436 (KT984005)
SR33	MH266145	*Halomonas gomseomensis* M12 (AM229314)
SR1	MH266146	*Halomonas janggokensis* FMH54 (KX821765)
28-C-6	MH266147	*Halomonas stenophila* N8 (MG563245)
A-10-2	MH266148	*Halomonas ventosae* NRS2HaP1 (LT221212)
L30	MH266149	*Idiomarina abyssalis* MCCC:1A05090 (KM407705)
L30-F	MH266150	*Idiomarina homiensis* MCCC:1A05917 (KM407707.1)
26-F-6	MH266151	*Idiomarina loihiensis* GSL 199 (CP005964)
26f-6	MH266152	*Idiomarina ramblicola* R22 (NR_025806)
L30B	MH266153	*Idiomarina salinarum* MCCC:1A02680 (KM407738)
D14-37	MH266154	*Lysobacter concretionis* Ko07 (NR_041003)
G-6-2	MH266155	*Marinobacter adhaerens* NIOSSK56#15(KY604871)
38CL	MH266156	*Marinobacter algicola* VSW110 (KC534310)
14Df	MH266157	*Marinobacter aquaticus* M6-53 (LT714149)
D-4-4	MH266158	*Marinobacter flavimaris* D6028 (FJ161304)
17-F-15	MH266159	*Marinobacter guineae* M3B (AM503093)
24fL	MH266160	*Marinobacter nanhaiticus* EAR19 (KU320883)
28-C-8	MH266161	*Marinobacter pelagius* KJ-W13 (JQ799110)
27-E-1	MH266162	*Marinobacter persicus* M9B (NR_109110)
D17-1	MH266163	*Marinobacter salsuginis* 11WSA1 (LN794817)
F-7	MH266164	*Marinobacter sediminum* UDC408 (HM031994)
709	MH266165	*Marinobacterium lutimaris* AN9 (NR_116590)
16SF	MH266166	*Microbulbifer salipaludis* SM-1 (NR_025232)
1-5-E	MH266167	*Microbulbifer taiwanensis* CC-LN1-12 (NR_108519)
D26	MH266168	*Modestobacter marinus* AL27 (KU258224)
R14	MH266169	*Palleronia marisminoris* 221-F2 (KJ638254)
SR14FL	MH266170	*Pararhodobacter aggregans* D1-19 (NR_115018)
18RL	MH266171	*Planococcus maritimus* Y67 (KU601234)
M28-R	MH266172	*Planococcus rifietoensis* M8 (CP013659)
L43-F	MH266173	*Pseudoalteromonas issachenkonii* KMM 3549 (CP011030)
L11-R	MH266174	*Pseudomonas psychrotolerans* Pp1 (MH233970)
23-B-5	MH266175	*Pseudoruegeria aquimaris* SW-255 (NR_043932)
M6RL	MH266176	*Rheinheimera pacifica* NBRC 103167 (NR_114230)
6FL	MH266177	*Rheinheimera tangshanensis* RB-213 (JQ361154)
13-B-7	MH266178	*Roseovarius mucosus* DFL-24 (NR_042159)
D-6-6	MH266179	*Roseovarius nubinhibens* SM25 (LT600603)
12-D-7	MH266180	*Roseovarius pacificus* 81-2 (NR_043564)
6-F-3	MH266181	*Roseovarius tolerans* EL-164 (KP723471)
20-B-2	MH266182	*Sediminimonas qiaohouensis* YIM B025 (EU878004)
4-C-4	MH266183	*Thalassospira profundimaris* WP0211 (NR_042766)
6-D-7	MH266184	*Wenzhouxiangella marina* 4S-CH-S3-s2 (MG264256)
M34FL	MH266185	*Winogradskyella arenosi* R60 (NR_041689)

### Analysis of the bacterial communities by DGGE fingerprinting

**Figure 1:**
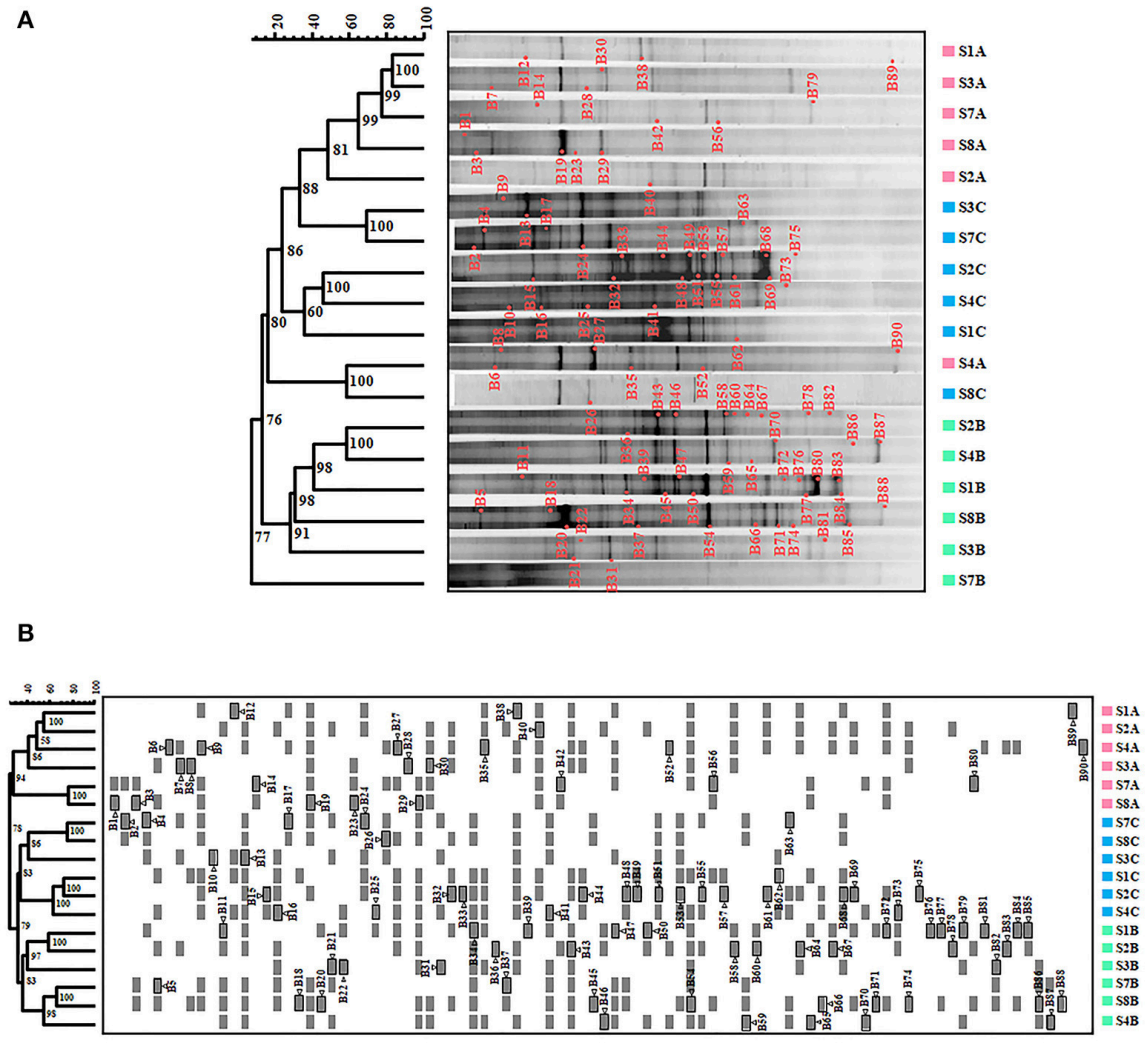
Bacteria in Rambla Salada analyzed by DGGE. **(A)** Pearson coefficient-based analysis. **(B)** Dice coefficient-based analysis. The *scale bar* indicates the percentage of similarity. *Numbers in nodes* represent the cophenetic correlation coefficient values. *Triangles followed by gray shaded squares* bands re-amplified and sequenced to conduct the phylogenetic study. *S1A* to *S8A*, sampling sites in June 2006; *S1B* to *S8B*, sampling sites in February 2007, and *S1C* to *S8C*, sampling site in November 2007.

DGGE fingerprint of samples were compared by using FPquest software. According to the intensity of the bands, the Pearson's coefficient based dendrogram (Figure 1A) showed two clusters with a 15% similarity level between them, indicating a low relationship between the two groups of bacteria. The first cluster includes the samples corresponding to June 2006 and November 2007 and the second cluster includes all the samples from February 2007.

Nevertheless, we obtained different results by using the Dice's coefficient, which is based on the presence or absence of bands. As shown in Figure 1B, we found two clusters with 36% similarity, including the June 2006 samples in the first one and the February and November 2007 samples in the second one. Samples from the upwelling zone (S7 and S8) in the Dice‘s dendrogram were grouped together during all three seasons studied.

### Phylogenetic analysis of the DNA sequences of the DGGE bands

**Figure 2:**
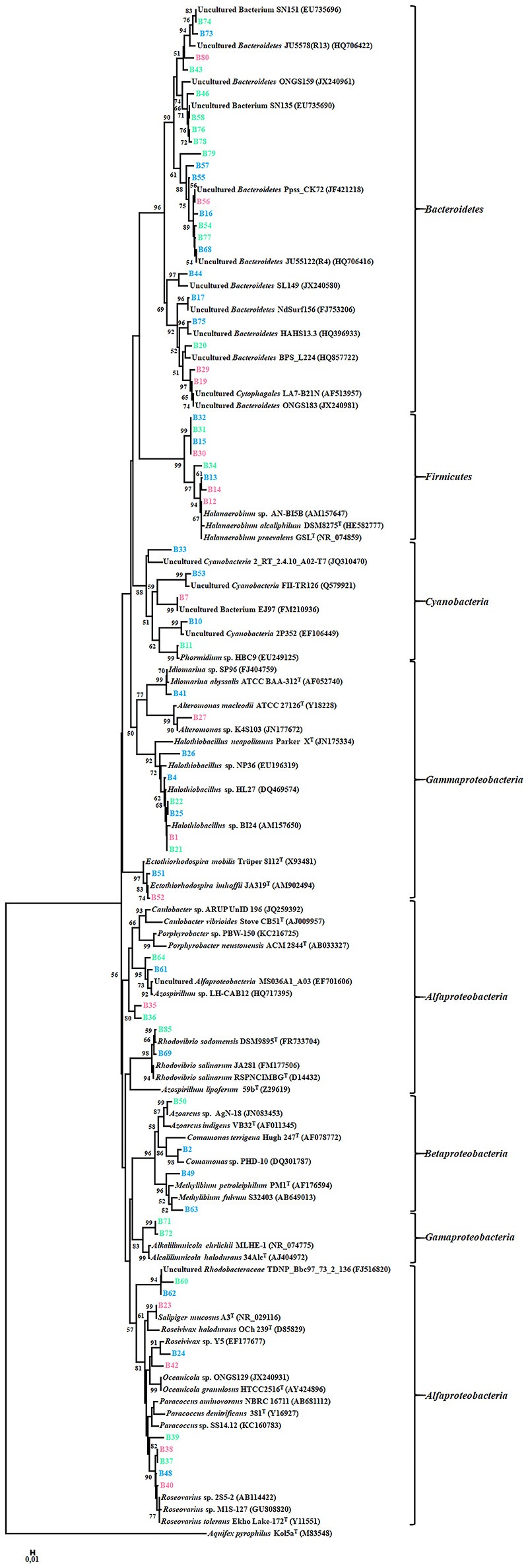
Neighbor-joining phylogenetic tree showing the relationships between the 67 bacterial sequences from the DGGE bands and the most similar sequences retrieved from the GenBank/EMBL/DDBJ database. The scale bar indicates 0.01% divergence. Bootstrap values over 50% are shown in nodes.

**Figure 3:**
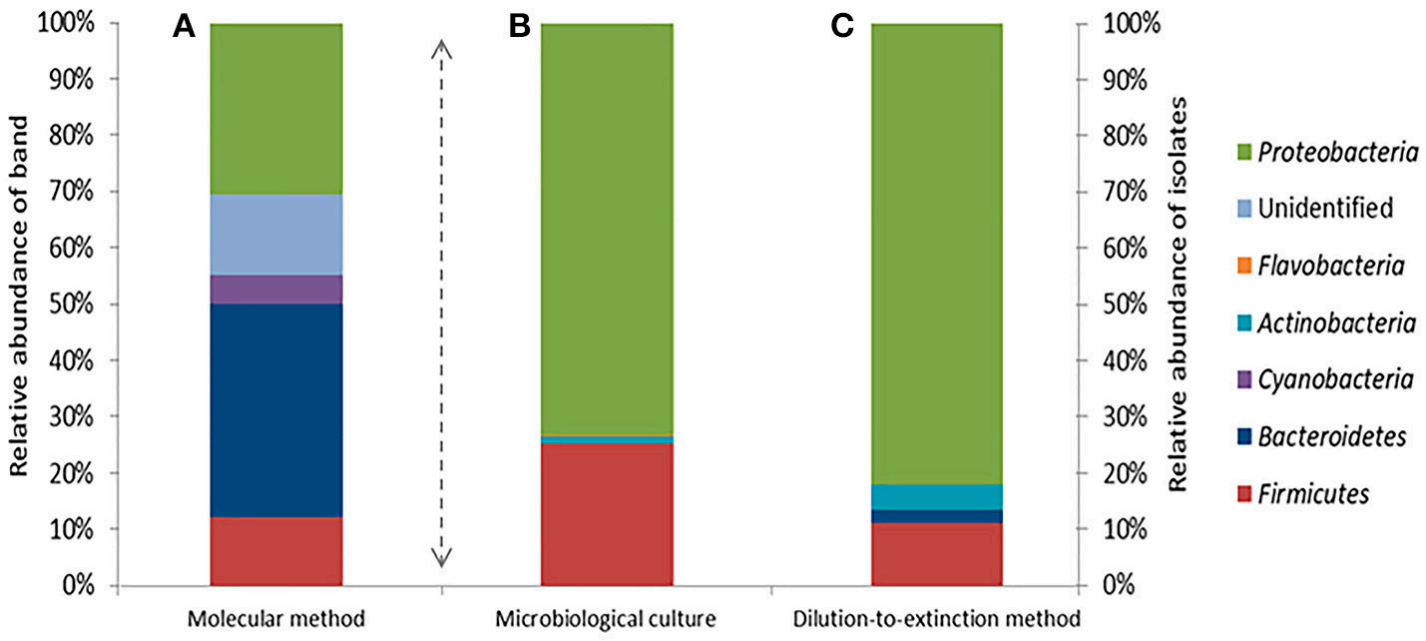
Bacterial diversity determined by analysis of the DGGE bands **(A)**, standard microbiological culture-dependent methods **(B)** (Data from Luque et al., 2014) and dilution-to-extinction method **(C)**.

A total of 67 DGGE bands were successfully reamplified and sequenced (around 500 bp each) from the 90 band classes detected. The identification of phylogenetic neighbors was carried out by the BLASTN (Altschul et al., 1997) program against the GenBank/EMBL/DDBJ database containing type strains with validly published prokaryotic names and representatives of uncultured phylotypes. The results are shown in Table 3. Clustering was determined using the neighbor-joining, maximum-parsimony, and maximum-likelihood algorithms giving the three, similar topologies and bootstrap values. The neighbor-joining phylogenetic tree (Figure 2) shows four main clusters, corresponding to the phyla *Bacteroidetes, Proteobacteria, Firmicutes, and Cyanobacteria*. Within the phyllum *Proteobacteria*, we found four groups, corresponding to *Alpha-, Beta-*, and *Gammaproteobacteria* classes. Figure 3 shows the bacterial diversity found by molecular techniques (DGGE) (a) in comparison to that identified by us in a previous work (Luque et al., 2014) in the same habitats, using classical culture techniques (b) and by a dilution-to-extinction approach (c). The relative abundance of identified sequences during the three seasons in Rambla Salada indicated that *Bacteroidetes* (39.73%) was the most abundant phylum with 46.07, 37.42, and 35.72% of relative abundance percentage in June 2006 and February and November 2007, respectively, followed by *Proteobacteria* (28.43%), showing higher proportion in samples taken in November 2006 (30.23%), and *Firmicutes* (8.23%) and *Cyanobacteria* (5.14%) with the highest proportion in samples taken in February 2007 (9.73 and 9.13%, respectively). A group of unidentified bacteria was also detected. All the sequences related with *Bacteroidetes* were identified as uncultured bacteria, while *Proteobacteria* included sequences related with species belonging to different genera, such as, *Idiomarina, Alteromonas, Halothiobacillus, Ectothiorhodospira, Caulobacter, Porphyrobacter, Azospirillum, Rhodovibrio, Azoarcus, Comamonas, Methylibium, Alkalilimnicola, Salipiger, Roseivivax, Oceanicola, Paracoccus*, and *Roseovarius*.

**Table 3:** 16S gene sequences obtained from the bands in DGGE and percentages of identity with their closest relatives.

**Band no**.	**GenBank acc. no**.	**Closet relatives and acc. no**.	**Identity (%)**
B1	MH252018	*Halothiobacillus* sp. XI15 (KT716396)	99
B2	MH252029	*Comamonas* sp. PHD-10 (DQ301787)	97
B4	MH252015	*Halothiobacillus* sp. XI15 (KT716396)	100
B7	MH252009	Uncultured Bacterium EJ97 (FM210936)	100
B10	MH252010	Uncultured *Cyanobacteria* 2P352 (EF106449)	98
B11	MH252011	*Phormidium* sp. HBC9 (EU249125)	99
B12	MH252006	*Halanaerobium alcaliphilum* DSM8275^T^ (HE582777)	99
B13	MH252004	*Halanaerobium* sp. AN-BI5B (AM157647)	99
B14	MH252005	*Halanaerobium* sp. AN-BI5B (AM157647)	95
B15	MH252001	*Halanaerobium* sp. AN-BI5B (AM157647)	94
B16	MH251989	Uncultured *Bacteroidetes* Ppss_CK72 (JF421218)	96
B17	MH251994	Uncultured *Bacteroidetes* NdSurf156 (FJ753206)	99
B19	MH251998	Uncultured *Cytophagales* LA7-B21N (AF513957)	99
B20	MH251996	Uncultured *Bacteroidetes* BPS_L224 (HQ857722)	98
B21	MH252019	*Halothiobacillus* sp. XI15 (KT716396)	100
B22	MH252016	*Halothiobacillus* sp. XI15 (KT716396)	99
B23	MH252036	*Salipiger mucosus* A3^T^ (NR_029116)	99
B24	MH252037	*Roseivivax* sp. Y5 (EF177677)	95
B25	MH252017	*Halothiobacillus* sp. HL27 (DQ469574)	100
B26	MH252014	*Halothiobacillus* sp. BI24 (AM157650)	94
B27	MH252013	*Alteromonas* sp. Disko Bay 11 (FJ581928)	99
B29	MH251997	Uncultured *Bacteroidetes* ONGS183 (JX240981)	99
B30	MH252002	*Halanaerobium* sp. AN-BI5B (AM157647)	94
B31	MH252000	*Halanaerobium* sp. AN-BI5B (AM157647)	99
B32	MH251999	*Halanaerobium* sp. AN-BI5B (AM157647)	94
B33	MH252007	Uncultured *Cyanobacteria* 2_RT_2.4.10_A02-T7 (JQ310470)	94
B34	MH252003	*Halanaerobium praevalens* GSL^T^ (NR_074859)	95
B35	MH252024	*Caulobacter* sp. ARUP UnID 196 (JQ259392)	94
B36	MH252025	*Porphyrobacter* sp. PBW-150 (KC216725)	94
B37	MH252041	*Roseovarius* sp. 2S5-2 (AB114422)	98
B38	MH252040	*Roseovarius* sp. M1S-127 (GU808820)	99
B39	MH252039	*Paracoccus* sp. SS14.12 (KC160783)	94
B40	MH252043	*Roseovarius tolerans* Ekho Lake-172^T^ (Y11551)	99
B41	MH252012	*Idiomarina* sp. SP96 (FJ404759)	98
B42	MH252038	*Oceanicola* sp. ONGS129 (JX240931)	95
B43	MH251980	Uncultured *Bacteroidetes* Ppss_Ma285 (JF421166)	99
B44	MH251993	Uncultured *Bacteroidetes* SL149 (JX240580)	92
B46	MH251981	Uncultured Bacterium SN135 (EU735690)	97
B48	MH252042	*Roseovarius* sp. 2S5-2 (AB114422)	99
B49	MH252030	*Methylibium fulvum* S32403 (AB649013)	96
B50	MH252028	*Azoarcus* sp. AgN-18 (JN083453)	98
B51	MH252020	*Ectothiorhodospira imhoffii* JA319^T^ (AM902494)	99
B52	MH252021	*Ectothiorhodospira imhoffii* JA319^T^ (AM902494)	99
B53	MH252008	Uncultured *Cyanobacteria* FII-TR126 (Q579921)	97
B54	MH251990	Uncultured *Bacteroidetes* JU55122(R4) (HQ706416)	98
B55	MH251987	Uncultured *Bacteroidetes* JU55122(R4) (HQ706416)	99
B56	MH251988	Uncultured *Bacteroidetes* Ppss_CK72 (JF421218)	97
B57	MH251986	Uncultured *Bacteroidetes* Ppss_CK72 (JF421218)	95
B58	MH251982	Uncultured Bacterium SN135 (EU735690)	99
B60	MH252034	Uncultured *Rhodobacteraceae* TDNP_Bbc97_73_2_136 (FJ516820)	99
B61	MH252023	*Azospirillum* sp. LH-CAB12 (HQ717395)	96
B62	MH252035	Uncultured *Rhodobacteraceae* TDNP_Bbc97_73_2_136 (FJ516820)	99
B63	MH252031	*Methylibium fulvum* S32403 (AB649013)	94
B64	MH252022	Uncultured *Alfaproteobacteria* MS036A1_A03 (EF701606)	96
B68	MH251992	Uncultured *Bacteroidetes* JU55122(R4) (HQ706416)	99
B69	MH252027	*Rhodovibrio sodomensis* DSM9895^T^ (FR733704)	99
B71	MH252032	*Alkalilimnicola ehrlichii* MLHE-1 (NR_074775)	94
B72	MH252033	*Alkalilimnicola ehrlichii* MLHE-1 (NR_074775)	94
B73	MH251978	Uncultured Bacterium SN151 (EU735696)	99
B74	MH251977	Uncultured Bacterium SN151 (EU735696)	99
B75	MH251995	Uncultured *Bacteroidetes* HAHS13.3 (HQ396933)	99
B76	MH251983	Uncultured Bacterium SN135 (EU735690)	99
B77	MH251991	Uncultured *Bacteroidetes* JU55122(R4) (HQ706416)	97
B78	MH251984	Uncultured Bacterium SN135 (EU735690)	98
B79	MH251985	Uncultured *Bacteroidetes* BPS_H527 (HQ857687)	98
B80	MH251979	Uncultured *Bacteroidetes* JU5578(R13) (HQ706422)	94
B85	MH252026	*Rhodovibrio salinarum* JA281 (FM177506)	98

### Analysis of diversity indexes

The average number of bands obtained in the DGGE per sample were 25, with a minimum of 14 bands in one sample from the upwelling zone (S8) taken in June 2006 and a maximum of 36 bands from the same zone (S8) taken in February 2007. The diversity and richness of the bacterial communities depended on the sampling season and showed average *Rr* index values ranging from 41.36 ± 25.95 to 74.14 ± 38.31 (Table 4) with high average richness-index values (*Rr* > 30) in the three seasons.

**Table 4:** Diversity indexes of bacterial communities in Rambla Salada at the three sites and sampling seasons.

**Sampling season**	**Range-weighted richness (*Rr*)**	**Functional organization (*Fo*)**	**Shannon-Weaver (*H'*)**	**Simpson's (*SI'*)**
June-06	41.36 ± 25.95^*^	59.52%^*^	2.22 ± 0.34^*^	0.13 ± 0.07^*^
February-07	74.14 ± 38.31	53.81%	2.60 ± 0.36	0.11 ± 0.06
November-07	58.73 ± 26.86	52.26%	2.63 ± 0.24	0.09 ± 0.02
LSD^‡^	0.0365	0.0128	0.0385	0.0251

* *Significant differences among the three sampling seasons*.

‡ *Least-significant difference at p < 0.05*.

The Shannon-Weaver index values (*H'*) were 2.22 ± 0.34, 2.60 ± 0.36, and 2.63 ± 0.24 for the June 2006, February 2007, and November 2007 samples respectively. The Simpson index (*SI'*), which represents dominance and is inversely proportional to the Shannon-Weaver index, were 0.13 ± 0.07, 0.11 ± 0.06, and 0.09 ± 0.02 respectively. ANOVA analysis (*p* < 0.05) revealed that there were significant differences in the diversity indexes from one season to another (Table 4).

**Figure 4:**
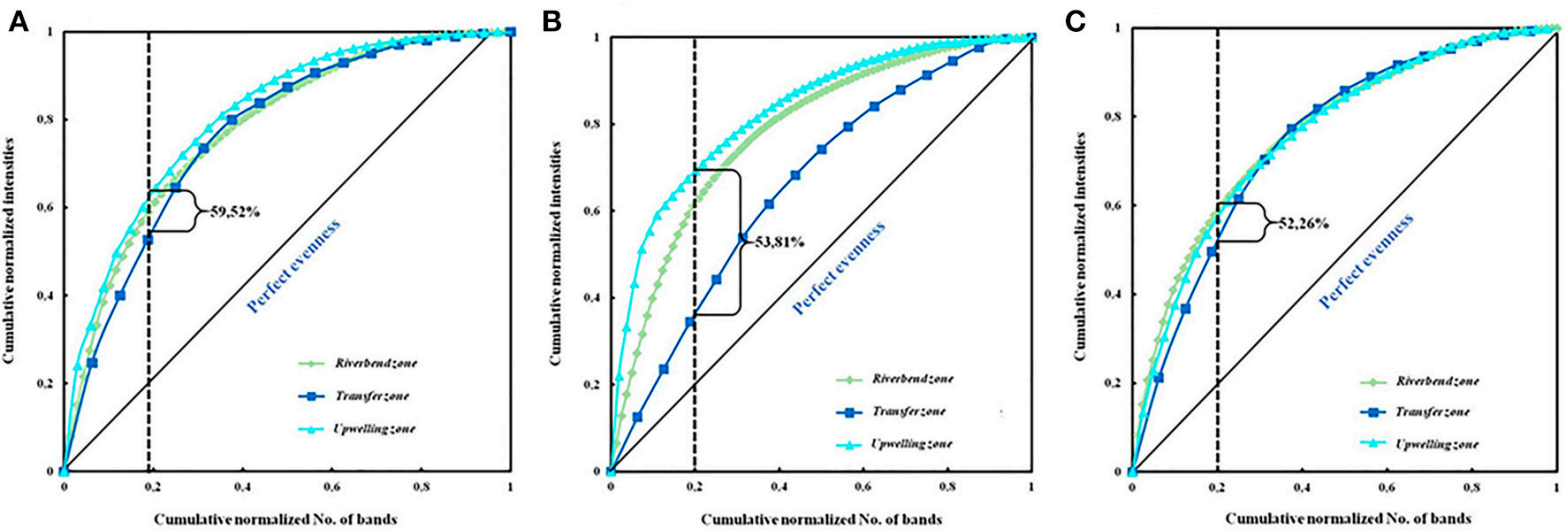
Pareto-Lorenz distribution curves based on the DGGE fingerprints of the bacterial community in the different sampling zones in June 2006 **(A)**, February 2007 **(B)**, and November 2007 **(C)**. The *vertical lines* at the 0.2 *x*-axis are plotted to determine the Pareto values (Fo).

The functional organization of the bacterial communities was carried out using the *Fo* index (Table 4) and the Pareto-Lorenz distribution curve (Figure 4). In the samples corresponding to June 2006, 20% of the bands showed *Fo* values of 60.50, 55.04, and 63.02% in the riverbed, river-transfer and upwelling zones respectively, and the average value of the cumulative band intensities being 59.52% (Figure 4A). Twenty per cent of the bands detected in the February 2007 samples represented 60.55, 35.77, and 65.13% (53.81% on average) of the accumulative band intensities (Figure 4B), and another 20% of the bands represented 60.55, 52.29, and 55.96% of the accumulative band intensities (52.26% on average) in the November 2007 samples (Figure 4C).

### Relationships between the composition of bacterial communities and environmental variables

DCA analysis were carried out to determine whether our data were unimodal or linear. DCA analysis showed that the data (2.841) exhibited an unimodal or lineal response to the environmental variables (Lepš and Šmilauer, 2003), so we decided to apply a CCA analysis. Table 5 shows the eigen values, the cumulative percentage variance in species data and the cumulative variance in the species-environment relationship along the three axes of the CCA analysis.

**Table 5:** Summary of CANOCO results.

**Axes inertia**	**1**	**2**	**3**
Eigenvalues	0.277	0.277	0.168
Specie-environment correlations	0.943	0.973	0.930
Cumulative percentage variance of species data	9.7	17.7	23.6
Cumulative percentage variance of species-environment relation	41.2	75.0	100.0

**Figure 5:**
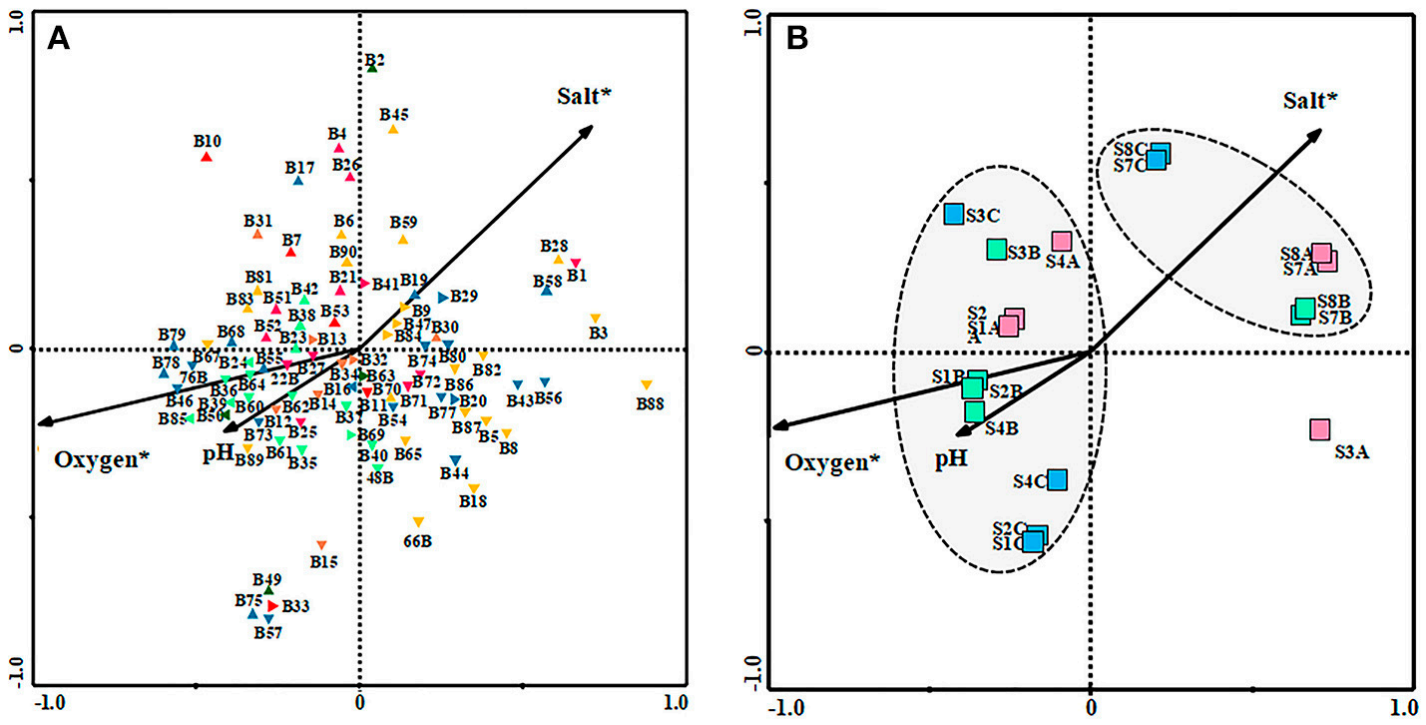
Canonical correspondence analysis (CCA) ordination diagram (biplot) of bacterial species with environmental variables [salt, pH and oxygen] **(A)** and of them with the site and sampling season [*SA* 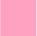 (June 2006), *SB* 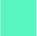 (February 2007), *SC* 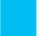 (November 2007). (*S1, S2*, and *S4*: Riverbend zone; *S3*: River-transfer zone; *S7* and *S8*: Upwelling zone)] **(B)**. Environmental variables are indicated as *arrows*. Environmental variables marked with asterisks are significant (*p* < 0.05). 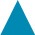
*Bacteroidetes*; 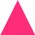
*Gammaproteobacteria*; 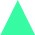
*Alfaproteobacteria*; 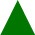
*Betaproteobacteria*; 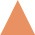 *Firmicutes*; 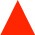
*Cyanobacteria*; 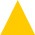
unidentified Bacteria.

Based on the 5% level in a partial Monte Carlo permutation test, the value for oxygen and salinity were significant (*P* < 0.05), providing 75 and 41.2%, respectively, of the total CCA explanatory power. Therefore, the data concerning the environmental factors contributing to the model were ranked in the following order: oxygen, salinity, and finally pH. Species environment correlation for the three axes was more than 0.93, suggesting that bacterial community were strongly correlated with these environmental factors. Figure 5 shows the influence of the environmental variables upon the diversity of bacterial community in the three seasons studied (Figure 5A) and also in relation to the sampling site (Figure 5B). Figure 5A, in which each environmental variable is represented by an arrow, the projection of any given taxon along an axis shows the level of the variable where the taxon is most abundant. CCA analysis showed a positive correlation with salinity on members belonging to the phylum *Bacteroidetes*, as well as *Gammaproteobacteria* class. Most of the uncultured bacteria also correlated positively with this environmental factor. Nevertheless, all the bacteria related to *Alpha-* and *Betaproteobacteria* class and phylum *Firmicutes* showed a positive correlation with oxygen and pH and negative with salinity. Finally, the phylum *Cyanobacteria* were less influenced by the environmental variables.

As seen in Figure 5B, in relation with the location of samples there were two different groups: group A, that included the upwelling zone samples (S7 and S8), showing a positive correlation with salinity and negative correlation with oxygen and pH, and group B, that included riverbed zone and transfer zone samples, showing a positive correlation with pH and oxygen. S3A sample, from the river-transfer conduit, taken in June 2006, is completely different from all the other samples analyzed and thus has not been taken into consideration in our interpretation of the results.

### Quantitative analysis of the microbial community as a whole and its bacterial component

**Figure 6:**
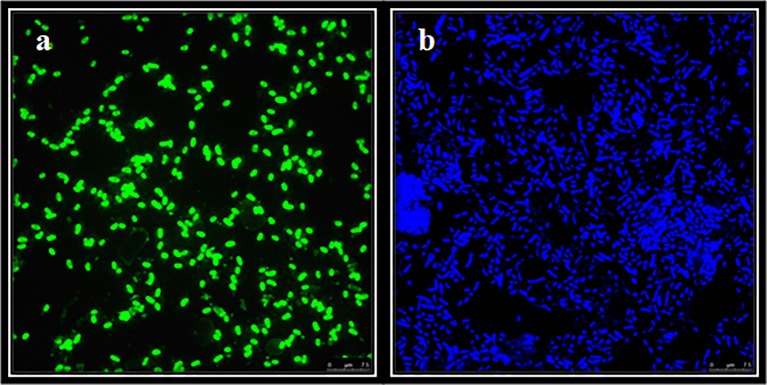
CARD-FISH of samples from Rambla Salada. FITC (total bacteria) counts from riverbed zone in February 2007 **(A)** and DAPI (total population) **(B)**. Image of bacterial cells visualized with a Leica TCS-SP5 CLSM. *Scale bars*, 7.5 μm.

Total microbial cells in Rambla Salada detected with DAPI staining were 6.1 × 10^8^, 6.7 × 10^8^, and 7.1 × 10^8^ cells/ml in February 2007, November 2007, and June 2006, respectively (CFU/ml) (Figure 6B). CARD-FISH, using universal probes for the *Bacteria* domain, allowed us to know that the bacteria population in Rambla Salada were 3.9 × 10^8^ in June 2006, 4.3 × 10^8^ in November 2007, and 4.8 × 10^8^ cells/ml in February 2007. Figure 6A shows a photograph of bacterial cells hybridized with universal bacterial probe (Eub338-HRP-FITC) in samples from riverbed zone in February 2007.

## Discussion

Microbial diversity studies has progressed increasingly since the inclusion of molecular techniques that allow to obtain the fingerprinting based on the 16S ribosomal RNA gene analysis, such as the Denaturing Gradient Gel Electrophoresis (DGGE). DGGE fingerprinting is a useful molecular tool due to number of bands and their intensities are related to diversity (Muyzer et al., 1993). Additionally, clustering and ordination methods can include environmental parameters that help to evaluate the impact of different factors on the community composition and structure (Besemer et al., 2005). Nevertheless, in spite of DGGE has significant limitations that should be considered like the possible bias introduced through the DNA extraction, PCR amplification, selection of universal primers and different number of rRNA gene copies (Neufeled and Mohn, 2006) the technique is still used to get an idea of the predominant bacterial population in a habitat (Tang et al., 2016; Yin et al., 2016; Garofalo et al., 2017; Huang et al., 2017; Panosyan et al., 2018). Moreover, DGGE technique permit use the FPquest software to determine several diversity indexes as we explained below.

In this work we studied the bacterial community of Rambla Salada by DGGE and by dilution-to-extintion culture and we compare these data with those obtained by our group with classical culture media (Luque et al., 2012b). Moreover, we determine the correlation of geographical location, season and physic-chemical parameters (oxygen, salinity, and pH) in the bacterial community.

The results from the DGGE fingerprint analyzed by FPquest software revealed time differences (sampling period) in the bacterial community. Dendrogram showed in Figure 1A, based on band intensities (Pearson's coefficient) clustered together samples gathered in June 2006 and November 2007 while samples taken in February 2007 were not related. In contrast, Dice's coefficient (Figure 1B) showed a clear partition of the three sampling periods in the bacterial community in Rambla Salada. As shown in Figure 1A, the diversity profile of bacteria obtained in June 2006 differed significantly from the ones taken in February and June 2007, however, the presence-absence and intensity of the bands were similar for all of these periods. Thus, UPGMA group method carried out with Pearson's coefficient was more sensitive to intensity relative variations of the bands (Huys and Swings, 1999). More conservative rates, as Dice's similarity coefficient (Schwalbach et al., 2005; Hewson et al., 2006a,b) are recommended for genetic fingerprinting like DGGE technique. In spite of the use of a variety of similarity rates, different results in the samples clustering, Pearson and Dice coefficients revealed differences in the bacterial community, depending on the sampling time period. Nevertheless, no relationship could be established between the sample type and a specific taxon.

We can conclude that sample type (soil or watery sediment) or sampling area does not seem to affect the bacterial clustering, with the exception of samples from the upwelling area (S7 and S8) that were clustered together in the different sampling period, as showed in the dendrogram obtained by the Dice index. The upwelling zone is a sulfurous saline water pool with particular characteristics due to the high amount of chlorides and the great sulfate proportion from plaster. This water pool stays constant along the year and it has a regular salt concentration, which might be the reason that the biodiversity found in this area was analogous but different of the other sampling sites (Table 1).

The statistical study carried out over the different sampling areas and periods using various diversity indexes, confirmed the relation between the microbial communities found and the sampling periods, being June 2006 community the one with greatest difference. Regarding *Rr*, February and November 2007 had the highest richness values (74.14 ± 38.31 and 58.73 ± 26.86, respectively (Table 4). *Rr* values above 30 are typical in microbial diverse environments, according to Marzorati et al. (2008).

When applying Shannon-Waver (H') diversity index (Shannon and Weaver, 1963), which take the number of species present in the study area (species richness) and the number of individual of every species into account, similar results were reported. In this case, February and November 2007 were the seasons with the highest diversity values (2.60 ± 0.36 and 2.63 ± 0.24, respectively); these values were above 2.5, indicating that a high bacterial diversity was present in Rambla Salada. Luque et al. (2014) obtained similar results analyzing the bacterial diversity using traditional culture-dependent methods in the same areas and time periods.

Simpson index (*SI*) was applied to determine the species dominance within a sample, is based on the number of species and their abundance. Dominance was the lowest in November 2007 (0.09 ± 0.02) and the highest in June 2006 (0.13 ± 0.07). These results confirm that dominance decreases concomitantly with an increase in diversity (Magurran, 1996).

The highest richness and diversity values were detected in samples taken from riverbed zone in February and November 2007 with a low salt and high oxygen concentrations. The high richness and diversity values were probably related to these two environmental factors as confirmed by using CANOCO. In fact, in this analysis, salinity and oxygen were the most significant environmental factors that affected the distribution and composition of the bacterial community. Jiang et al. (2007) demonstrated that the salinity was the dominant factor influencing the composition and community structure of the bacteria population in a hypersaline lake located in Tibet, where the sample with highest salt concentration exhibited the least diversity. Similar results have been previously reported by different authors (Benlloch et al., 2002).

Bacterial community uniformity was determined by Pareto-Lorenz (PL) curves, showing the majority of the analyzed samples, medium *Fo* values (ranging from 40 to 60%) (Figure 4 and Table 4), indicating that the communities were balanced and could, therefore, potentially deal with changing environmental conditions and preserve their functionality (Marzorati et al., 2008). Samples taken in June 2006 and February 2007, however, seem to have similar biogeographical patterns and *Fo* index above 60%, indicating that the bacterial community in this zone and time period was more specialized, due to having a few dominant species, whilst the others were only represented by a few cells (Marzorati et al., 2008).

Ninety DGGE band classes in total were detected, and 67 of them were sequenced. Identified sequences were affiliated to phyla *Bacteroidetes, Proteobacteria* (*Alfa-, Beta-*, and *Gammaproteobacteria* classes), *Firmicutes*, and *Cyanobacteria* (Figure 2 and Table 3). Different authors have reported that in saline and hypersaline habitats, the most abundant groups of cultivable bacteria are affiliated to *Bacteroidetes, Proteobacteria*, and *Firmicutes* phyla (Benlloch et al., 2002; Mouné et al., 2003; Dong et al., 2006; Jiang et al., 2006, 2007; Maturrano et al., 2006; Mesbah et al., 2007; Mutlu et al., 2008; Hollister et al., 2010; Makhdoumi-Kakhki et al., 2012). The cultured bacteria present in soils and sediments, taken around the world from saline and hypersaline environments, mainly belong to the phylum *Proteobacteria* (Hollister et al., 2010; López-López et al., 2010; Swan et al., 2010; Nemergut et al., 2011; Luque et al., 2014). Nevertheless, our results showed differences between the bacterial community determined by molecular techniques and those obtained by culture-dependent methods (dilution-to-extinction and classical methods) (Luque et al., 2014). Maturrano et al. (2006) and (Panosyan et al., 2018) also showed differences between the biodiversity studied by molecular methods and classical methods in different hypersaline habitats.

In this study, 39.73% of the relative abundance of the total bacterial community was affiliated to uncultured taxa belonging to phylum *Bacteroidetes*, indicating that it was the dominant group. These features agreed with the results previously obtained by Makhdoumi-Kakhki et al. (2012), who reported that 59% of the identified sequences in Aran-Bidgol Lake (Iran) were affiliated to phylum *Bacteroidetes*, corresponding the 40% to *Salinibacter* genus. *Salinibacter* has been described as the most abundant taxon in different solar salterns located in Mallorca and Alicante, Spain (Antón et al., 1999, 2000; Rosselló-Mora et al., 2003) and in Çamalti saltern, the biggest artificial marine solar saltern in Turkey (Mutlu and Güven, 2015), as well as in different hypersaline lakes (Maturrano et al., 2006; Mesbah et al., 2007; Mutlu et al., 2008; Makhdoumi-Kakhki et al., 2012). Nevertheless, we could not detect *Salinibacter* in soil or watery sediments in Rambla Salada. All the sequences affiliated to *Bacteroidetes* were identified as non-cultivated species. Therefore, salt concentrations of Rambla Salada (1.1–15.8%, w/v) may not be suitable for *Salinibacter* to growth. Phylum *Bacteroidetes* was widely spread in every sampling area and season, even in low, medium and high salt concentrations samples. These results agreed with those obtained using CANOCO software, in which most of the sequences affiliated to *Bacteroidetes* were in salinity axis (Figure 5A). *Bacteroidetes* dominance in saline and hypersaline environments has been previously reported (Antón et al., 1999, 2000; Rosselló-Mora et al., 2003; Makhdoumi-Kakhki et al., 2012) and its presence increases with the increase of salinity (Benlloch et al., 2002; Demergasso et al., 2004, 2008; Jiang et al., 2006).

*Proteobacteria* was the second most abundant phylum, including members of *Alpha-, Beta-, and Gammaproteobacteria* classes. Wu et al. (2006) demonstrated that when salinity increase, the relative abundance of *Betaproteobacteria* class members decreases, but the relative abundance of *Apha-* and *Gammaproteobacteria* taxa increases. These results agreed with different studies carried out in continental waters (Böckelmanna et al., 2000; Brümmer et al., 2000), estuaries (del Giorgio and Bouvier, 2002; Kirchman et al., 2005; Henriques et al., 2006; Zhang et al., 2006), solar salterns (Benlloch et al., 2002), and a DGGE study in the soda saline crater lake from Isabel island, in the eastern tropical Pacific coast of Mexico (Aguirre-Garrido et al., 2016). In our study, the sequences affiliated to class *Alpha-* and *Betaproteobacteria* showed a negative correlation with salinity and positive with oxygen and pH, whilst class *Gammaproteobacteria* was positively related to salinity. So, the correlation with salinity is the same that above studies in the case of sequences affiliated to *Beta*- and *Gammaproteobacteria*, however the *Alphaproteobacteria* showed a different correlation with salinity. Nevertheless, Langenheder et al. (2003) demonstrated that *Alpha-, Beta-*, and *Gammaproteobacteria* classes were more abundant in low saline conditions. Jiang et al. (2007) reported that *Betaproteobacteria* members were the most abundant class, within the *Proteobacteria* phylum, in different lakes located in Tibet, northeast China, with high salt concentration, and no variations of the relative abundance of *Alpha-* and *Gammaproteobacteria* members along the salinity gradients were found.

The relative abundance of phylum *Firmicutes* represents 8.23% of the total bacterial community in Rambla Salada. Different authors reported that *Firmicutes* taxa only represents 11 to 25% of the phylotypes detected in most of the saline and alkaline lakes (Scholten et al., 2005; Jiang et al., 2006; Mesbah et al., 2007). In a study carried out in Aran-Bidgol Lake (Iran), 6% of the relative abundance of taxa belonged to phylum *Firmicutes* (Makhdoumi-Kakhki et al., 2012). Nevertheless, in a study carried out in a saline-alkaline soils in Ararat Plain (Armenia) that combined DGGE and culture-dependent method a dominance of *Firmicutes* populations inhabited by moderately halophilic bacilli belonging to the genera *Halobacillus, Piscibacillus, Bacillus*, and *Virgibacillus* was found (Panosyan et al., 2018). In Rambla Salada, the DGGE bands identified as *Firmicutes* members were negatively related with salinity, except the B30 band (94% of identity with *Halanaerobium*). Jiang et al. (2007) corroborated our results since they reported that the abundance of *Firmicutes* taxa was high in low salt concentration areas but low in high concentration areas.

The relative abundance of phylum *Cyanobacteria* in Rambla Salada was 5.14%. This percentage was also agree with Makhdoumi-Kakhki et al. (2012) who reported that 8% of clones retrieved from Aran-Bidgol Lake (Iran) were affiliated to this phylum.

On the other hand, by using classic culture methods, we determined that the bacterial population in Rambla Salada was mainly affiliated to phylum *Proteobacteria* and *Firmicutes* (72.5 and 25.8%, respectively) (Luque et al., 2014). The genus *Halomonas* and *Marinobacter* within phylum *Proteobacteria* were the predominant (40 and 13% respectively), whilst *Bacillus* genus was the most prevalent within phylum *Firmicutes*. In this work using DGGE, the *Halomonas* genus was not detected; instead this taxon is the easiest isolated in hypersaline environments. However, *Halomonas* was detected by DGGE in Rambla Salada using specific primers (Oueriaghli et al., 2014).

Extinction culturing methods have evolved from most probable number (MPN) techniques, where highest positive dilutions of MPN or dilution series provide enrichments, or even pure cultures, of abundant but fastidious bacteria that are often undetected by conventional culturing methods (Yang et al., 2016). Nutrient composition also determines the profile of the microbes that can be recovered on artificial media. Excessive nutrient concentrations are atypical in natural environments and can inhibit growth of bacteria adapted to oligotrophic conditions when they are transferred to high nutrient concentrations (Sait et al., 2002; Janssen, 2009). Adaptations to low substrate concentrations in natural environments likely have been a major factor contributing to bacterial unculturability. To address this limitation, novel media modifying the key parameters that simulate the environment (e.g., amount of nutrients, nutrient composition, the presence of trace elements, and pH) have been designed for optimized bacterial growth. Furthermore, nutrient-rich media favoring colonies of fast-growing bacteria can inhibit colony formation of slower growing species and negatively affects the recovery of difficult-to-culture organisms. Therefore, low nutrient media, combined with long incubation periods at relatively low temperatures and pH adjustments can allow bacteria with slow growth rates to form colonies; a great range of bacteria obtained with this approach have proved to be novel organisms (Kenters et al., 2011; Yang et al., 2016).

Dilution-to-extinction approach in combination with S3 low-nutrient-medium (Sait et al., 2002, 2006) and long incubation periods at 25°C, allowed us to obtain 354 isolates, after re-isolation the positives wells in R2A medium plates. The results showed an increment in the cultivability percentages (Figure 3) compared to those obtained by classical isolation techniques. By the dilution-to-extinction technique we obtained isolates belonging to *Proteobacteria* (81.9 %), *Firmicutes* (11.3%), *Actinobacteria* (4.5%), and *Bacteroidetes* (2.2%), while Luque et al. (2014) found the same phyla but different percentages (72.5, 25.8, 1.4, and 0.3, respectively). Using dilution-to-extinction technique, we were able to isolate more bacteria belonging to phyla *Actinobacteria* and *Bacteroidetes* than those obtained by classical culture media. However with both techniques the main isolated genera were the same, *Halomonas* and *Marinobacter. Cyanobacteria* members, detected by DGGE, were not isolated by any of both methods, probably due to culture conditions used.

Using dilution-to-extinction method, we obtained 9 isolates that showed less than 97% 16S sequence identity and may well represent new taxa. Recently, one of this species belonging to the genus *Blastomonas, B. quesadae* has been characterized (Castro et al., 2017).

To determine the number of bacteria at Rambla Salada, we carried out a direct count using CARD-FISH and universal bacterial probe. The results showed the highest counts in February 2007 (4.8 × 10^8^ cells/ml). These results agreed with the ones obtained by DGGE and diversity indexes. The percentage of bacteria found in the different sampling areas and seasons ranged from 54.3 to 78.9% of the total prokaryotic population. These results were similar to that found in a hypersaline deposit in the Canadian High Arctic (Niederberger et al., 2010). The cultivable bacteria counted in Rambla Salada ranged from 10^6^ to 10^7^ CFU/ml (Luque et al., 2014), which only represents 1% of the total bacterial population detected by molecular methods.

In conclusion, the methods combination used in this study allow us to demonstrate a reliable description of the bacterial populations in the different sampling areas at Rambla Salada, and to find new uncultured taxa so far. Moreover, we shown the correlation of environmental variables with the dominance of several phylum and we demonstrate that the predominant taxa found by DGGE aren't correlated with those isolated by dilution to extinction techniques. Our study highlights and confirms the relevance of this habitat as a diversity reservoir described previously using culture-dependent approach (Luque et al., 2014).

## Author contributions

NO performed the experimental DGGE techniques and statistical analysis. DC performed the experimental dilution-to-extinction techniques. IL performed the design of dilution-to-extinction technique and comparative study with culturable methods. VB performed the design of DGGE study and analysis of DGGE results. FM-C performed the design of DGGE study and dilution-to extinction technique and executed the analysis of the results and drafting of the manuscript.

### Conflict of interest statement

The authors declare that the research was conducted in the absence of any commercial or financial relationships that could be construed as a potential conflict of interest.
